# Breaking the barrier: disruption of bacterial biofilms using microwave radiation

**DOI:** 10.3389/fcimb.2025.1670237

**Published:** 2025-11-18

**Authors:** Harita Ben, Harshita Agarwal, Bharat Gurnani, Aman A. Pradhan, Arani Ali Khan, Neha Jain

**Affiliations:** 1 Department of Bioscience and Bioengineering, Indian Institute of Technology, Jodhpur, Rajasthan, India; 2 Centre of Excellence-AyurTech, Indian Institute of Technology, Jodhpur, Rajasthan, India; 3 Department of Electrical Engineering, Indian Institute of Technology, Jodhpur, Rajasthan, India

**Keywords:** biofilm disinfection, microwave irradiation, antimicrobial resistance, medical device sterilization, extracellular matrix

## Abstract

Biofilms are microbial consortia encased in the extracellular matrix that pose severe threats in healthcare and environmental settings due to their resistance to antimicrobials and their role in persistent infections. These structured communities colonize medical devices (e.g., catheters, implants) and contribute to nosocomial infections. Critically, biofilm-laden medical waste acts as a reservoir for multidrug-resistant pathogens and facilitates horizontal gene transfer, perpetuating antimicrobial resistance (AMR). Improper disposal risks environmental contamination, enabling pathogens to infiltrate water systems, soil, and food chains, exacerbating public health crises. Conventional methods like chemical disinfection or UV treatment often fail to dismantle biofilms, leaving viable pathogens to disseminate. In the present work, we have established the use of microwave radiation as an effective alternative strategy for pre-disposal sterilization of *Escherichia coli* UTI89 biofilm on different surfaces. In our results, 15 minutes of microwave exposure significantly reduced cell viability by up to 95% and regrowth potential by up to 25% of *E. coli* UTI89 biofilms formed on coverslips and catheter-mimicking surfaces. Microwave-treated biofilms showed marked structural disruption and increased membrane permeabilization, as confirmed by FE-SEM and CLSM analyses. These findings highlight microwave radiation as a promising strategy for efficient pre-disposal sterilization and mitigating environmental risks associated with biofilm-derived pathogens in healthcare waste. These findings support the use of microwave exposure as an innovative approach for sterilizing medical waste and controlling biofilm-associated pathogens, aligning with current global efforts to identify sustainable alternatives for infection control. Overall, our results indicate that microwave radiation could be implemented as an innovative strategy for effective pre-disposal sterilization, reducing the risks of environmental AMR dissemination from medical waste, and curbing biofilm-derived pathogens in landfills and water systems. We firmly believe that implementing our approach in conjunction with current modalities in clinical workflows could reduce device-related infections and help alleviate the burden of AMR.

## Introduction

Biofilms are complex, three-dimensional microbial communities in which microorganisms, typically bacteria, are embedded within a self-produced matrix of extracellular polymeric substances (EPS) (Beloin et al., 2008). This matrix provides structural integrity and protection, and is composed of extracellular nucleic acids, polysaccharides, protein polymers, lipids, and water (Flemming et al., 2016). The transition from the planktonic (free-floating) state to a sessile, biofilm-associated lifestyle is a highly regulated adaptive response that enhances microbial survival under environmental stress, including desiccation, nutrient limitation, antimicrobial agents, and host immune defense (Hall and Mah, 2017).

The complex architecture and components of EPS act as a barrier and protect the residing bacteria from environmental stress (Flemming et al., 2016). Biofilms have shown resistance and adaptations to extreme temperature, pH, high salinity, and pressure (Agarwal et al., 2025). The EPS matrix exhibits selective permeability, allowing the nutrients and signalling molecules, while impeding the penetration of antimicrobial compounds (Flemming et al., 2016). These structured communities possess 10-1, 000-fold higher antibiotic resistance than planktonic cells, attributed to matrix-mediated diffusion barriers, metabolic heterogeneity, and persister cell subpopulations (Ciofu et al., 2022). Biofilms can be found on biotic surfaces, such as teeth plaque and kidney stones, and on abiotic surfaces, including medical devices such as catheters, pacemakers, and industrial pipelines (Agarwal et al., 2025). In healthcare settings, biofilms colonize approximately 80% of indwelling medical devices, including catheters, prosthetic joints, and ventilators, contributing to 65% of nosocomial infections globally (da Silva et al., 2021). In India, the escalating antimicrobial resistance (AMR) crisis due to carbapenem-resistant *Klebsiella pneumoniae*, methicillin-resistant *Staphylococcus aureus* (MRSA), and uropathogenic *Escherichia coli* in 70% of intensive care units is exacerbated by biofilm persistence (Reports | Indian Council of Medical Research | Government of India). The associated economic burden is staggering, with biofilm-related infections prolonging hospital stays and increasing treatment costs (Frost et al., 2019). A critical yet not fully addressed facet of biofilm management lies in the pre-discarding sterilization of contaminated medical waste (Garvey, 2023). Biofilms on discarded devices harbour multidrug-resistant pathogens such as *E. coli*, MRSA, and *Pseudomonas aeruginosa*, which evade standard waste protocols, facilitating horizontal gene transfer and environmental persistence of resistance traits (Garvey, 2023). For instance, hospital effluents containing biofilm fragments have been linked to carbapenemase-producing *K. pneumoniae* in wastewater systems, posing cross-contamination risks (Garvey, 2023).

Conventional methods, including chemical disinfection, such as chlorine and quaternary-ammonium-based disinfectants, often achieve success in killing planktonic cells; however, they fail to dismantle the biofilms due to the EPS matrix, enabling viable pathogens to reseed clinical and/or ecological niches (da Silva et al., 2021). Other conventional medical waste treatment methods, such as pyrolysis vaporization, rotary kiln incineration, plasma incineration, chemical disinfection, and autoclaving, are widely used but come with significant limitations, including high capital costs, skilled labour requirements, residual chemical contaminants, emission of hazardous gases, equipment degradation, and poor efficacy against biofilm (Ghasemi and Yusuff, 2016; Ghodrat et al., 2017; Ahmad et al., 2019; Das et al., 2021; Bhatt et al., 2022). Recent advances in physical sterilization technologies, such as non-thermal plasma (NTP) and ultrasonic irradiation, demonstrate partial efficacy but face limitations in scalability, cost, or material compatibility (Mai-Prochnow et al., 2016). NTP targets biofilms by generating reactive oxygen species (ROS) and reactive nitrogen species (RNS); however, *P. aeruginosa* biofilms have recently been reported to exhibit partial survival post-NTP treatment due to matrix-mediated resistance (Kašparová et al., 2022). These high-power approaches often lack specificity toward EPS components, and their energy-intensive nature restricts deployment in decentralized or low-resource settings.

On the other hand, microwave irradiation presents a promising alternative with its dual thermal and non-thermal mechanisms. The thermal effect of microwaves differs from traditional, more time-consuming heat sterilization techniques, as they utilize the dielectric property of polar substances for rapid temperature rise (Zhao et al., 2023). In addition to dielectric heating, microwaves exert non-thermal effects through oscillating dipolar molecules and electromagnetic field interactions that may destabilize membrane potential, generate localized ROS, and affect protein conformation (Banik et al., 2003). Unlike chemical disinfectants, microwave treatment leaves no toxic residues, reduces ecological contamination, and aligns with sustainable waste management protocols (Bonez et al., 2013; Kollu et al., 2022). Prior studies have shown the effectiveness of microwaves, mainly over planktonic and minimal biofilm systems, in utilizing high-power microwaves (500–800 W) (Wang et al., 2024). The reported high power achieves partial biofilm disruption but risks substrate degradation, particularly in heat-sensitive polymers (Banik et al., 2003; Wang et al., 2024).

Here, we have utilized shorter exposure to microwave radiation for optimal disruption of biofilms by a uropathogenic *E. coli* strain UTI89. Operating at 2.45 GHz, a frequency within the license-free Industrial, Scientific, and Medical (ISM) band, this system offers ideal penetration and uniform dielectric heating of aqueous environments, without inducing excessive surface temperature gradients. We tested the impact of microwave radiation on *Escherichia coli* biofilms formed on glass coverslips and catheter mimics. Our results demonstrate that controlled microwave exposure leads to significant structural disintegration of the biofilm matrix with minimal thermal load. Our study highlights the translational potential of microwave technology as a residue-free, material-safe, and energy-efficient method for disinfection. We firmly believe that when integrated with current protocols, this approach could offer a scalable solution and substantially lower treatment-associated costs.

## Materials and methods

### Bacterial culture and biofilm preparation

For the preparation of *E. coli* UTI89 (ATCC, # 364106) biofilms, four-way streaking was performed from glycerol stocks in Luria Bertani (LB) broth, and the plates were incubated for 8 to 12 hours at 37°C. Isolated colonies from the plate were inoculated in 2–3 mL of LB media and incubated at 37°C for 12–14 hours at 200 rpm. After 14 hours, 3 mL of YESCA (yeast extract-Casamino Acids) media + 4% DMSO and 6 µL (2.6 × 10^8^ CFU/mL) of primary culture were added to 12-well plates with coverslips in a slant position. For biofilm over a catheter-mimic, catheter tubing (2 mm length) was cut and placed in individual wells of a sterile 12-well plate. The plate was inoculated with the bacterial suspension and incubated under static conditions at 25°C for 4 days to facilitate biofilm development. For subsequent experimental procedures, both coverslips and catheter segments with established biofilms were carefully retrieved using sterile forceps to avoid disruption of the biofilm structure.

### Biofilm exposure to microwave radiation

The coverslip and catheter mimics with grown biofilm were carefully removed from the well plate using forceps, placed on butter paper, and exposed to microwave radiation with varying intensities and exposure times. We used a thermal gun (Fluke 561) to check the microwave temperature.

### Biofilm exposure to ultraviolet radiation

The coverslip with grown biofilm was taken out from the well plate carefully with forceps, placed on butter paper, and exposed to UV radiation in a biosafety cabinet (1300 series A2- ThermoScientific) for 20 minutes.

### Biofilm exposure to dry heat (conventional method)

To assess the effect of temperature alone on biofilm integrity, coverslips with established biofilms were aseptically removed from the culture wells using sterile forceps and placed on sterile butter paper. The samples were then subjected to controlled heat exposure in a dry incubator (ThermoScientific) at two defined conditions: 45°C for 10 minutes and 56°C for 15 minutes. These temperatures were selected based on the maximum thermal readings recorded during microwave treatment at corresponding durations. This control experiment was performed to decouple thermal effects from non-thermal (e.g., electromagnetic) effects of microwave exposure.

### Sample preparation for assays

Untreated and treated biofilms over coverslips were resuspended in 2 mL 1X phosphate buffer saline (PBS) buffer, pH 7.4. Biofilm was dislodged from the coverslip by repeated pipetting, and then the solution was homogenized using a probe sonicator (Labman) with 5% power for one cycle of 5-second pulses. Homogenized samples were further used for different assays.

### Bacterial growth measurement

The untreated and treated coverslips with biofilm were resuspended in 2 mL of LB broth to regrow at 200 rpm and 37°C, and the optical density of the samples was quantified at 0 and 8 hours at 600 nm wavelength using a microtiter plate reader (Max Spectra M2e-Molecular Devices). The experiment was performed in three independent biological replicates.

### Cell viability quantification using MTT assay

An MTT (3-(4, 5-dimethylthiazol-2-yl)-2, 5-diphenyl tetrazolium bromide) assay was performed to measure cell viability in untreated and microwave-treated biofilm samples, as well as biofilm samples treated with dry heat in an incubator (Stindlova et al., 2024). 200 µL of samples were incubated with 0.1 mg/mL MTT at 37°C for 60 minutes in the dark. After incubation, samples were centrifuged at 10, 000 rpm for 10 minutes to allow the formazan to settle. The formazan formed by the enzymatic reduction of MTT in the biofilm in the untreated and treated samples was dissolved in 200 µL DMSO (dimethyl sulfoxide), and quantified by measuring the absorbance at 570 nm in a microplate reader (SpectraMax M2e-Molecular Devices). The experiment was performed in three independent biological replicates.

### Biofilm biomass quantification using crystal violet assay

The crystal violet assay was performed to measure biofilm biomass in untreated and microwave-treated biofilm samples. The untreated and microwave-treated biofilm samples were stained with 0.05% crystal violet for 3 minutes. The excess stain was washed with water, and the samples were allowed to dry overnight in an incubator at 37°C. The dried biomass was resuspended in 33% glacial acetic acid, and the absorbance was measured at 595 nm in a 96-well plate using a microplate reader (SpectraMax M2e-Molecular Devices) (Mazumder et al., 2024).

### Hydrophobicity measurement using ANS fluorescence assay

For measuring the hydrophobicity of the biofilm matrix, we have used 200 µL of untreated and microwave-treated biofilm samples and incubated them with 10 µM ANS (8-anilino-1-naphthalenesulfonic acid) at 37°C for 15 minutes in the dark (Zhao et al., 2023). After incubation, samples were transferred to a black 96-well plate for spectroscopic analysis. Fluorescence emission spectra were recorded using a microplate reader (SpectraMax M2e-Molecular Devices) with an excitation of 380 nm and with emission spectra recorded across the range of 400–600 nm in 10 nm increments. The experiment was performed in three independent biological replicates.

### Biofilm ROS quantification using DFCH-DA

For measuring the reactive oxygen species (ROS) in biofilm, we have used 200 µL of untreated and microwave-treated biofilm samples and incubated them with 50 µM DCFH-DA (2’, 7’-dichlorodihydrofluorescein diacetate) at 37°C for 15 minutes in the dark (Zhang et al., 2022). Fluorescence intensity was measured using a microplate reader at 485 nm excitation and 530 nm emission using a microplate reader (Max Spectra M2e-Molecular Device). The experiment was performed in three independent biological replicates.

### Morphological characterization of biofilms using FESEM and confocal microscope

Biofilm samples, 200 µL of untreated, microwave-treated, and UV-treated biofilm suspensions were centrifuged at 12, 000 × *g* for 10 minutes at 4°C. The resulting pellets were fixed with 2% glutaraldehyde in 1× PBS and incubated overnight at 4°C. Following fixation, samples were rinsed with 1× PBS and dehydrated through a graded ethanol series (30%, 50%, 70%, 90%, and 100%), with 5-minute washes at each step (Mazumder et al., 2024).

For intact (unhomogenized) samples, biofilms grown directly on coverslips and catheter-mimic tubes were fixed with 2% glutaraldehyde without sonication, followed by a similar PBS rinse and ethanol dehydration protocol.

After dehydration, all samples were transferred to silica wafers and dried overnight in a desiccator at room temperature. The dried specimens were mounted onto aluminium stubs using carbon adhesive tape and sputter-coated with a thin layer of gold to enhance conductivity. Imaging was performed using a field-emission scanning electron microscope (Apreo 2S, Thermo Fisher Scientific) equipped with an Everhart–Thornley detector (ETD). Samples were visualized at an accelerating voltage of 10 kV and a magnification of 16, 000× to assess biofilm architecture, cell morphology, and membrane integrity. The experiment was performed in three independent biological replicates.

For confocal laser scanning microscopy, biofilms were cultured in 48-well plates with a coverslip. After four days, biofilms were gently removed from the well plate and treated with a microwave. The untreated and treated biofilm were then stained with SYTO 9 (5 μM final concentration in 1X PBS) and PI (Propidium Iodide) (30 μM final concentration in 1X PBS) and incubated for 30 minutes at 37°C in the dark, and FilmTracer™ SYPRO Ruby Red (200 μL), which labels extracellular protein components, was incubated for two hours at 37°C in the dark (Agarwal et al., 2025). The samples were rinsed gently with 1X PBS to remove excess dye. The biofilms were fixed using 2% glutaraldehyde for 2 hours at 4°C. After that, the biofilm sample was mounted over a cleaned 75 mm × 25 mm glass slide, where the *E. coli* UTI89 biofilms stayed between the glass slide and the coverslip. A 100X oil immersion objective (Numerical aperture 1.45) was used to focus the sample by using a laser scanning confocal microscope (FV1000 FLUOVIEW- Olympus). For both SYTO 9 and SYPRO Ruby Red dye staining, a CW-laser of 488 nm (Cobolt Skyra) was used as an excitation source; for PI staining, a CW-laser of 559 nm (Cobolt Skyra), with a power of 25 mW, was passed through acousto-optic tunable filters (AOTF), and then broadband single-mode fiber optics. Before detection by a photomultiplier tube (PMT), the fluorescence signal was passed through a grating-based band-pass filter (2 nm resolution). However, for the imaging of SYTO 9 dye, the emission bandpass was set between 500–600 nm. For SYPRO Ruby Red dye, the emission bandpass was kept in the range of 580–680 nm, and for PI, the emission bandpass was set in the range of 600–700 nm. After focusing, the sample was imaged sequentially for z-stack imaging with a gap of 0.3 microns for each set of experiments. Images were processed and analyzed using the free version of ImageJ software. Surface plots were generated using the 3D Surface Plot plugin from the z-stacks to assess structural changes in biofilm topography. Fiji image analysis software was used for splitting channels for live-dead imaging of biofilms. The CLSM experiment was performed using one biological replicate.

### Quantification of DNA in biofilm matrix using Qubit dsDNA HS assay kit

For estimation of total DNA content in biofilm samples before and after treatment, sample were centrifuged at 10, 000 rpm for 10 minutes at 25°C. The supernatant was used for DNA quantification using Qubit dsDNA HS Assay Kit (ThermoFischer Scientific). The sample dilution in buffer was performed as per the user guide (Pub.No. MAN0002326 C.0) provided by the manufacturer, and samples were analyzed using the Qubit fluorometer (ThermoFisher Scientific) after incubation. The experiment was performed in two independent biological replicates.

### Material integrity assessment for the catheter-mimic tube

The material integrity of the catheter-mimic tube following microwave exposure was assessed using a bubble immersion test. After treatment, one end of the catheter segment was securely sealed, and the other end was attached to a syringe. The catheter was then submerged in water, and air was purged from the syringe into the tube. The presence or absence of air bubbles was carefully observed to identify any leaks or structural deformity from microwave exposure. This experiment was designed to detect potential surface damage or perforation on the catheter material caused by the sterilization protocol.

### Data plotting and statistical analysis

Experimental results are presented as mean values derived from a minimum of three independent biological replicates, with error bars denoting the standard deviation (SD). Data visualization and graphical representations were generated using Origin Pro^®^ (version 9.0, licensed software), ImageJ (v1.53q, free version) and Fiji (Latest, free version). Statistical comparisons between experimental groups were performed using an unpaired t-test, with significance thresholds defined as follows: *p < 0.05, **p < 0.01, ***p < 0.001, ****p < 0.0001, and “ns” (not significant) for p ≥ 0.05. Origin Pro^®^ was utilized for nonlinear curve, ImageJ was used for 3D surface plot generation, while Fiji was used for live-dead image data analysis.

## Results

### Microwave generation and power optimization

**Figure 1:**
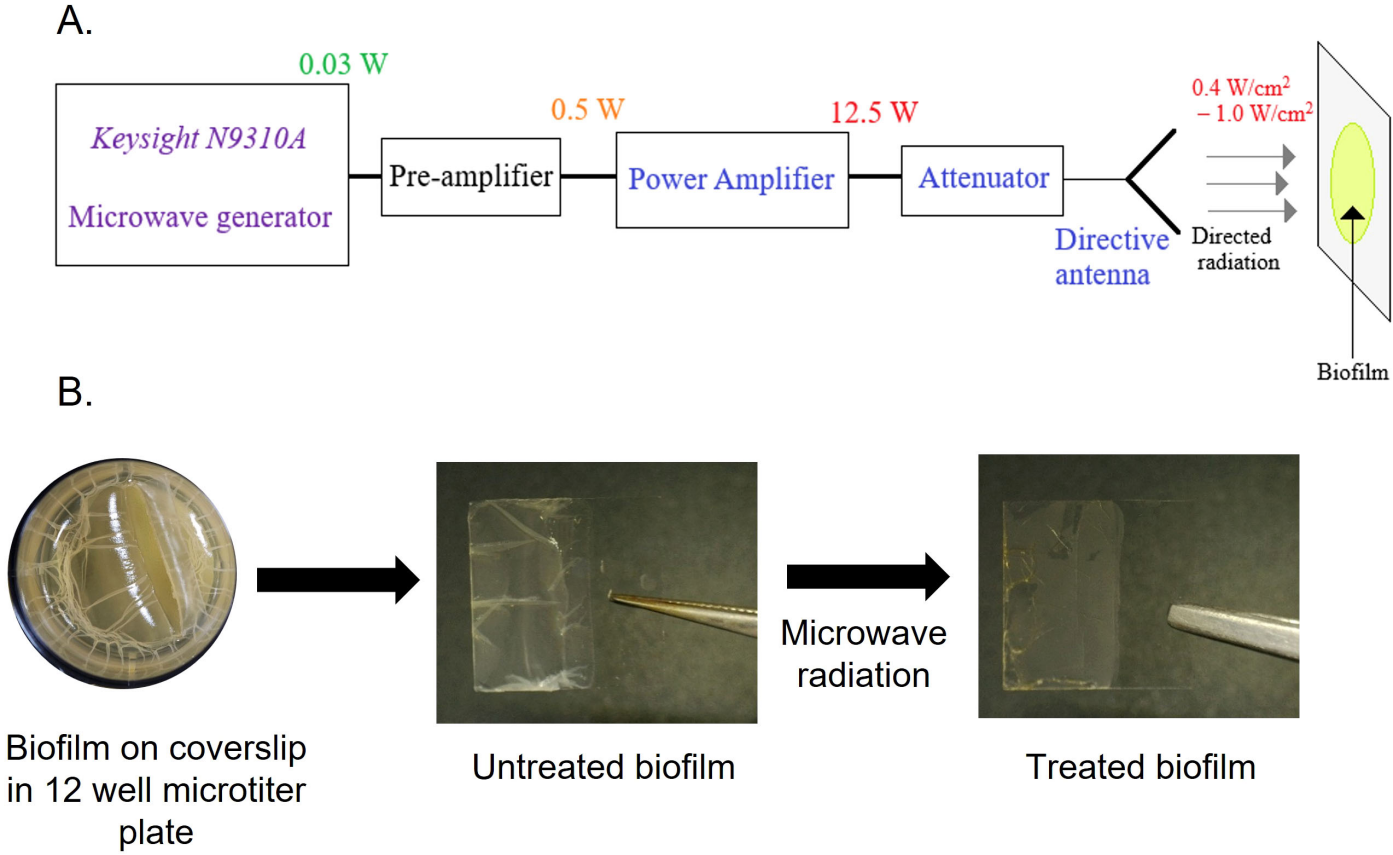
Microwave setup and procedure for microwave exposure to biofilm. **(A)** Schematic of microwave irradiation system (frequency, power settings, and sample stage). **(B)***E coli* UTI89 biofilm grown for four days over a coverslip at 25°C in a 12-well microtiter plate in YESCA + 4% DMSO broth. Coverslip with biofilm exposed to microwave radiation.

We first designed the setup for microwave generation. **Figure 1A** shows the block diagram of the experimental setup. The low-power microwave generator *Keysight N9310A* acts as the primary source of 2.45 GHz radiation. This low-power signal is then amplified using the pre-amplifier and Power Amplifier (PA) designed by the research group of the Indian Institute of Technology Jodhpur (Shukla et al., 2025). The maximum output power of the PA in Continuous Wave (CW) mode is 12.5 W. Finally, this amplified microwave power is fed to a directive antenna system to concentrate the radiation on the biofilm grown over coverslips and a catheter mimic. The variable attenuator placed between the amplifier and the antenna system regulates the radiation intensity. Fine-tuning of the power level is also possible at the output of the *Keysight N9310A* microwave generator. To observe the effects of microwave radiation on bacterial biofilms, initially, microwave radiation with intensities of 0.2 W/cm^2^, 0.4 W/cm^2^, and 1 W/cm^2^ was used with different exposure times (10–60 minutes) (**Figure 1B**; **Supplementary Figure 1A–C****).** From the preliminary results, the minimum required radiation intensity of 0.4 W/cm^2^ was finalized for effective destruction of biofilms at two different time points (10 and 15 minutes).

### Microwave treatment reduces the regrowth capacity and metabolic activity of *E. coli* UTI89 biofilm

**Figure 2:**
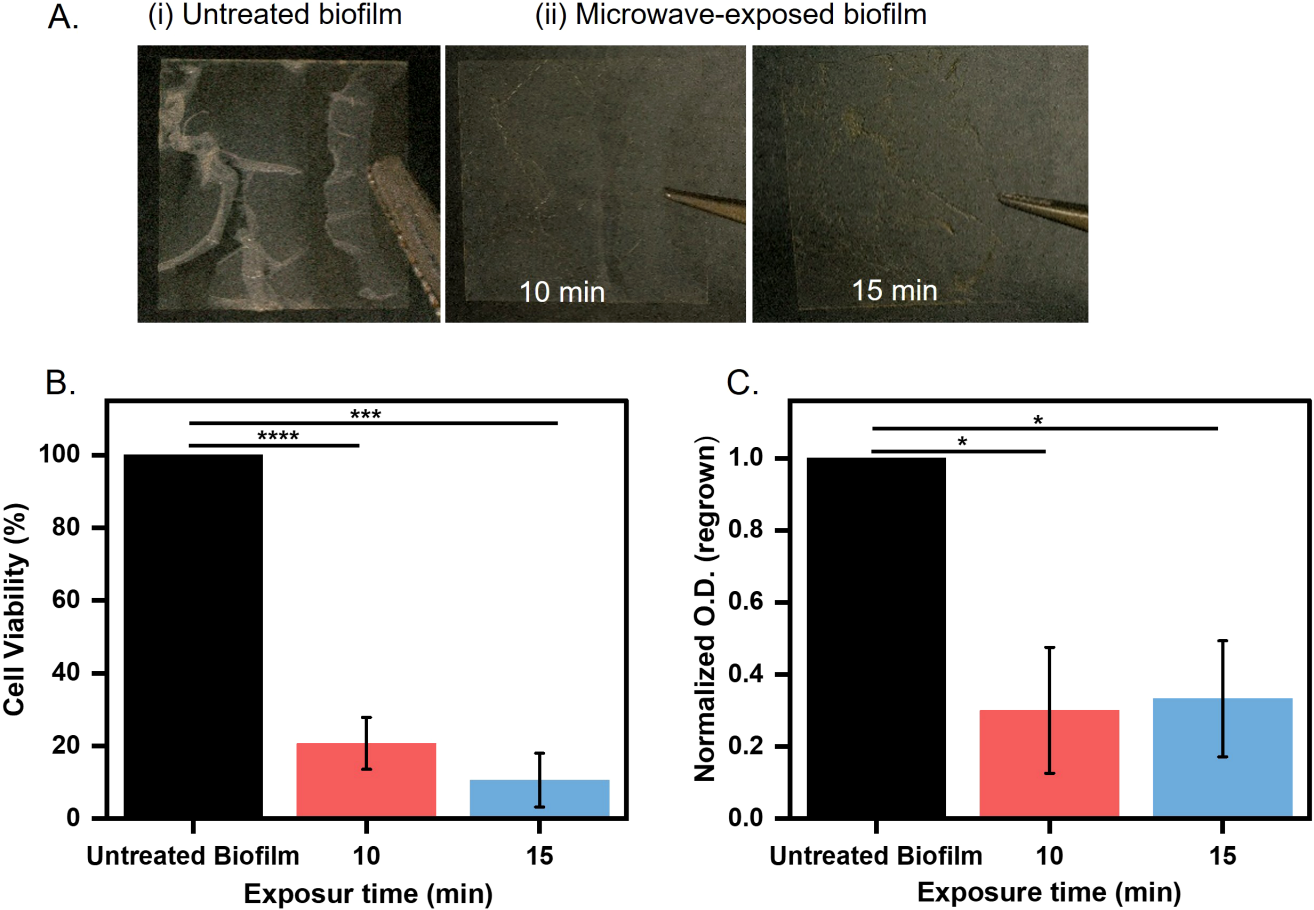
Biofilm grown over coverslip **(A)** Untreated and treated biofilms microwaved for 10 and 15 minutes at 0.4 W/cm^2^. **(B)** Microwaves exposed biofilms for 10 and 15 minutes at 0.4 W/cm^2^ assessed for cell viability by MTT assay (***p < 0.001, ****p < 0.0001). **(C)** Microwave-exposed biofilms at 0.4 W/cm² were quantified by measuring optical density at 600 nm after regrowth for 8 hours in LB broth at 37°C (*p < 0.05).

We initiated our studies by investigating the efficacy of microwave irradiation in destabilizing biofilm architecture and reducing bacterial viability. We prepared *E. coli* UTI89 biofilm in 12-well plates on a coverslip. One set of biofilms was treated with microwave radiation with an intensity of 0.4 W/cm^2^ for 10 and 15 minutes. After the treatment, we captured the images of the untreated and treated biofilm using a digital LCD microscope (TOMLOV). We observed an intact thick biofilm (untreated) on the coverslip, whereas the microwave-treated biofilm was visibly weak and fragile (**Figure 2A**). Microwave treatment leads to a moderate increase in temperature, which imposes thermal effects (Lv et al., 2019). Real-time thermography revealed temperature increases of 20.3°C (10 minutes) and 34.7°C (15 minutes) during irradiation (**Supplementary Figure 2A**), consistent with dielectric heating mechanisms reported for microbial disinfection (St et al., 2024; Kornsing et al., 2025). These thermal gradients align with prior studies demonstrating that sustained temperatures >40°C impair bacterial membrane integrity and protein function, leading to reduced viability and proliferation (Cebrián et al., 2017).

Thermal stress is directly correlated with metabolic activity and cell viability in biofilms (Beckwith et al., 2020). To understand the effect of microwave treatment on *E. coli* UTI89 biofilms, we quantified the metabolic activity of biofilm-embedded cells using the 3-(4, 5-dimethylthiazol-2-yl)-2, 5-diphenyltetrazolium bromide (MTT) assay, which measures dehydrogenase-mediated reduction of tetrazolium salts to formazan crystals (Stindlova et al., 2024). Biofilms from treated and untreated coverslips were incubated with MTT, and formazan precipitation was quantified spectrophotometrically. Microwave exposure resulted in a significant reduction in metabolic activity of *E. coli* UTI89 biofilms, with an 80% decrease observed after 10 minutes and a 95% decrease after 15 minutes compared to untreated controls (**Figure 2B**). These results indicate a strong suppression of metabolically active biomass (***p < 0.001 and ****p < 0.0001, respectively). We further aimed to determine the change in biofilm biomass following microwave exposure, which may result from physical disruption or detachment of the biofilm matrix. To assess total biofilm biomass, we performed crystal violet (CV) staining following microwave exposure. CV binds to cellular components and extracellular polymeric substances, allowing quantification of both viable and non-viable adherent biomass. This enabled us to evaluate structural integrity and biomass retention independently of metabolic activity, providing insight into the extent of biofilm disruption caused by microwave treatment. A ~20% and ~40% reduction in biofilm retention was observed after 10 and 15 minutes of microwave treatment, respectively, compared to untreated controls (**Supplementary Figure 2B**). This dual approach allows us to determine whether microwave treatment primarily inactivates cells (metabolic suppression without biomass loss) or disrupts the biofilm structure (biomass removal). In our study, both assays showed significant reductions, indicating that microwave exposure effectively reduces both viable cell numbers and overall biofilm integrity. The drastic decrease in metabolic activity led us to speculate that the cells in biofilms may have lost the capability to revive after microwave treatment. To assess this, the untreated and treated biofilms were regrown in optimal media for 8 hours. We observed a 25% reduction in regrowth for microwave-exposed samples as compared to untreated biofilms, which were able to grow again. The reduction in regrown capability was significant (*p < 0.05) (**Figure 2C**). This decline in cell population suggests permanent damage to cellular repair mechanisms, such as DNA replication or ATP synthesis pathways (Zhang et al., 2022). These results suggest that the antibiofilm effects of microwave exposure are not solely attributable to thermal mechanisms, indicating a significant non-thermal component in the disruption of biofilm integrity and metabolic activity. To evaluate the thermal contribution, biofilm samples were exposed to the maximum temperatures achieved during microwave treatment: 45°C for 10 minutes and 56°C for 15 minutes, in a controlled incubator. Under these purely thermal conditions, cell viability assays revealed reductions of ~35% and 40%, respectively, compared to unexposed controls (**Supplementary Figure 2C**). In contrast, microwave exposure induced substantially greater biofilm inactivation (80–95%), despite a similar temperature rise. These results strongly support the involvement of non-thermal effects, such as electromagnetic field-induced membrane permeabilization, or enhanced molecular agitation, which act synergistically with heat to disrupt biofilm structure and function beyond what can be achieved by temperature alone. These results prompted us to investigate the detailed impact of microwave radiation on cellular and matrix structure.

### Microwave treatment disrupts biofilm morphology and reduces matrix content

**Figure 3:**
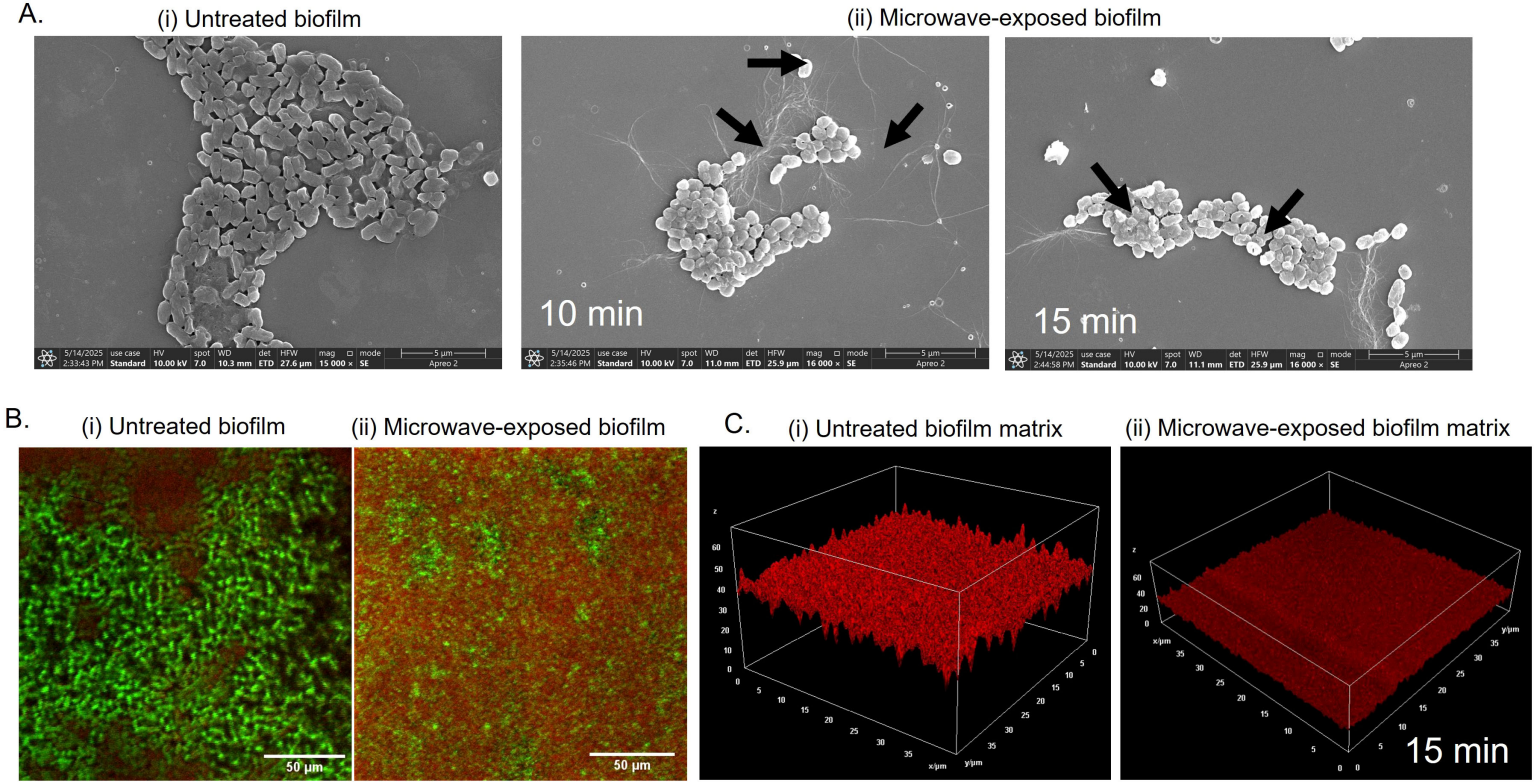
Biofilm cells and matrix disruption by microwave exposure **(A)** Microwave-exposed biofilm was imaged under FE-SEM to visualize changes in cell structure and matrix morphology. **(B)** For CLSM imaging, biofilm with coverslip grown in a 48-well microtiter plate for 4 days at 25°C in YESCA + 4% DMSO broth and stained prior to imaging with SYTO-9/PI (live/dead) and **(C)** FilmTracer™ SYPRO^®^ Ruby Red for matrix protein distribution.

Next, we visualized untreated and microwave-treated biofilms under field-emission scanning electron microscopy (FE-SEM) to understand the morphological changes induced by microwaves. Untreated *E. coli* UTI89 biofilms exhibited intact rod-shaped morphology with a layer of extracellular polymeric substance (EPS), characteristic of robust biofilm architecture (Hung et al., 2013) (**Figure 3A****; i)**. However, microwave irradiation induced severe damage to the cells, leading to a loss of structural integrity. As observed in **Figure 3A****; ii** cells displayed membrane perforations and collapsed ovoid forms. The cell distortion correlated with exposure time, reflecting progressive dielectric heating and electromagnetic stress-induced membrane destabilization (Barkhade et al., 2025). To ensure that the observed morphological changes were not artifacts of sample processing due to mild sonication, we performed FE-SEM analysis of untreated and microwave-treated samples processed without sonication. Untreated biofilm samples exhibited intact rod-shaped cells, characteristic of healthy *E. coli*. In contrast, microwave-exposed samples displayed ovoid forms with clear membrane disruptions and structural damage. The absence of morphological alterations in sonicated but non-irradiated controls indicates that the observed cellular damage is specifically attributed to microwave exposure and not due to the homogenization process (**Supplementary Figure 3A, B**). The cellular disintegration was further confirmed by assessing the viability of the untreated and microwave-exposed cells in the biofilm using live/dead staining. We utilized SYTO9 and PI (propidium iodide) to differentiate live (green) and dead (red) cells based on membrane integrity. SYTO 9 can permeate all cells, while PI preferentially stains membrane-compromised cells (Agarwal et al., 2025). Our confocal laser scanning microscopy (CLSM) data revealed intense SYTO 9 fluorescence in untreated biofilms (**Figure 3B****; i)**, with ~78% viable and healthy intact cells. Following post-microwave exposure (15 minutes), an increase in PI fluorescence was observed, indicating cells with compromised membranes. This suggests a significant increase in dead cell population to about 74%, confirming microwave-induced membrane permeabilization and cell death (**Figure 3B**; ii).

The main barrier in biofilms that protects the biofilm community is the extracellular matrix, which consists of protein polymers, polysaccharides, lipids, and extracellular DNA. Proteinaceous cell surface adhesins, flagella and pili components, and extracellularly secreted proteins significantly influence the attachment and stability of microbial communities by mediating cell-to-surface and cell-to-cell adhesion (Fong and Yildiz, 2015). The proteinaceous components interact with exopolysaccharides and extracellular nucleic acids and contribute to the 3D architecture and mechanical integrity of the biofilm (Flemming et al., 2016).

We wondered if the microwave has any effect on the proteinaceous components of the biofilm matrix. We deployed FilmTracer™ SYPRO^®^ Ruby Red, a matrix-protein specific dye, to analyze microwave-induced deformities in the matrix (Hochbaum et al., 2011). CLSM images of untreated biofilms revealed high fluorescence, indicating a continuous, homogenous matrix with a dense protein network (**Figure 3C**; i). However, microwave-exposed samples exhibited significantly low fluorescence, indicative of a fragmented and thin matrix with reduced protein content (**Figure 3C**; ii). Overall, our microscopy analysis suggests that microwaves disrupt the biofilm matrix with a reduction in matrix components and cause damage to the cell membrane, leading to metabolically inactive cells incapable of growing again (**Figures 2B, C****).**

### Microwaves alter physicochemical properties of biofilm

**Figure 4:**
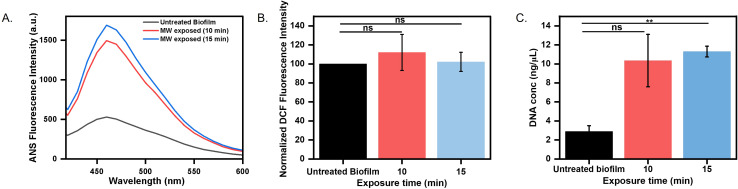
Biofilm physicochemical alteration estimation **(A)** Heat-induced hydrophobicity of untreated and microwaved-treated biofilm grown over a coverslip determined by ANS fluorescence intensity. **(B)** ROS production determined by DCF fluorescence intensity **(C)** Estimation of leaked DNA content in matrix after microwave exposure for 10 minutes and 15 minutes (ns: non-significant, **p < 0.01).

The pronounced reduction in the biofilm matrix after microwave exposure indicated that it may alter biofilm matrix hydrophobicity, a critical determinant of structural integrity. ANS (8-anilino-1-naphthalenesulfonic acid) is an environment-sensitive fluorophore, which is nonfluorescent in an aqueous environment but exhibits high fluorescence upon interaction with exposed hydrophobic regions. In our experimental setup, we observed low ANS fluorescence in untreated biofilms (**Figure 4A**), indicating an intact matrix with minimal exposure of the hydrophobic surface. In contrast, microwave-exposed biofilms exhibited a three-fold increase in fluorescence intensity, indicating exposure of the hydrophobic surface due to damage in the matrix (**Figure 4A**). Our findings correlate with previous studies that showed increased hydrophobicity to enhanced matrix porosity and permeability (Zhao et al., 2023).

Oxidative stress is a common cellular response to environmental perturbations (Čáp et al., 2012). To evaluate whether microwave-induced stress triggers oxidative responses in the biofilm, intracellular ROS levels were measured using Dichlorodihydrofluorescein Diacetate (DCFH-DA). It is a non-fluorescent probe, gets deacetylated and oxidized to the fluorescent compound DCF (dichlorofluorescein) in the presence of ROS, providing a reliable readout of oxidative stress levels within biofilms (Kim and Xue, 2020). We did not observe a significant difference in ROS production by untreated and microwave-treated biofilms (**Figure 4B**), indicating that microwave exposure did not substantially increase ROS production within biofilm cells. Our result corroborates with previous studies that have reported no effect of microwave exposure on ROS levels (Zhang et al., 2022). We could conclude that microwave exposure damages the biofilm in an oxidative-independent manner. We further estimated the total DNA content in the matrix, which could increase due to leakage from cell membrane damage. We observed a significant increase in DNA content when the biofilm was exposed to microwave irradiation at 0.4 W/cm² for 15 minutes, causing damage to membrane integrity. Our results align with previous reports, where they also found an increase in DNA release upon microwave exposure (Woo et al., 2000) (**Figure 4C**).

### Microwave treatment is efficient than conventional UV disinfection

**Figure 5:**
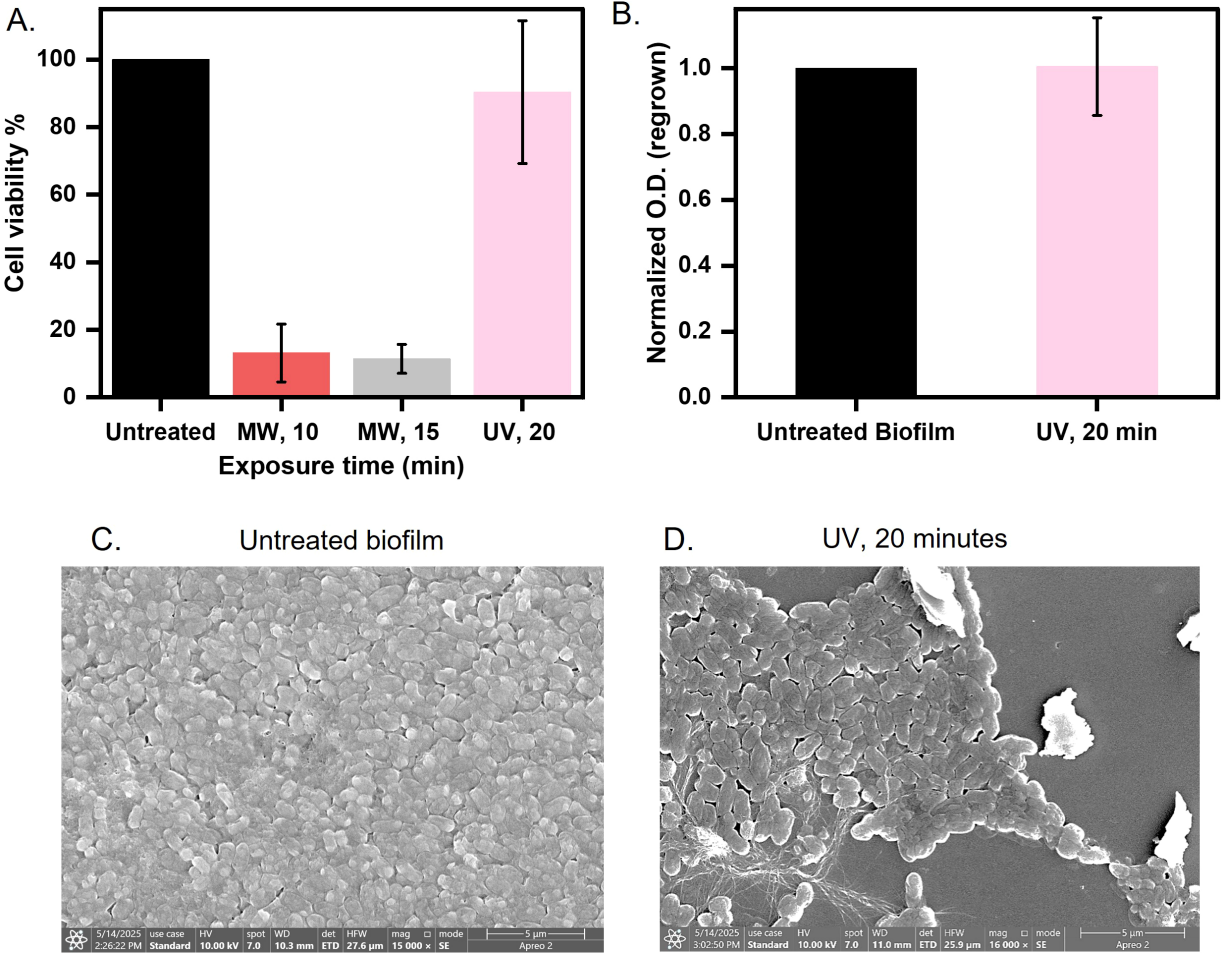
Comparison between microwave and UV exposure for biofilm disinfection **(A)** Biofilm over coverslip was exposed to microwaves at 0.4 W/cm^2^ for 10 and 15 minutes and UV for 20 minutes and assessed for cell viability by MTT assay. **(B)** A 20-minute UV-exposed biofilm was quantified at 600 nm after regrowing for 8 hours in LB broth at 37°C. **(C, D)** Morphology observed of untreated and 20-minute UV-exposed biofilm imaged using FE-SEM.

UV radiation is commonly used to kill bacteria in various settings (Bintsis et al., 2000). However, the effectiveness of UV on biofilms is less-known (Elasri and Miller, 1999). The EPS matrix blocks UV penetration and needs higher doses to inactivate biofilm (Argyraki et al., 2017). Therefore, we aimed to compare whether our strategy of microwave exposure could be a more effective approach than UV radiation for sterilizing biofilm-infected surfaces. We exposed biofilms under UV for 20 minutes and assessed the cell viability via MTT assay. We observed a negligible impact of UV radiation on biofilms, whereas microwaves reduced viability by 80% (10 minutes exposure) and 90% (15 minutes exposure) (**Figure 5A**). Regrowth assays in nutrient-rich media demonstrated that UV-treated biofilms proliferated equivalently to untreated ones (**Figure 5B**), and ANS fluorescence intensity was found to overlap with untreated biofilms but showed a three-fold increase after microwave exposure, indicating matrix protein denaturation and hydrophobic residue exposure (**Supplementary Figure 4**). FE-SEM imaging further differentiated the mechanism of disruption. UV-treated cells retained intact rod morphologies, similar to those of untreated cells (**Figures 5C, D**), consistent with photochemical DNA damage. In contrast, microwaves induced membrane perforations, cytoplasmic collapse, and ovoid cell shapes (**Figure 3A**(ii)); (Ahlawat et al., 2024). The better efficacy of microwaves stems from synergistic thermal (dielectric heating >40°C) and non-thermal (electromagnetic stress) mechanisms that disrupt membrane integrity and destabilizes the extracellular matrix (Zhao et al., 2023). In contrast, UV radiation relies on DNA damage, which is mitigated by biofilm EPS shielding and bacterial repair pathways. Microwave-induced hydrophobicity shifts and structural fragmentation impede nutrient retention and microbial recovery, critical for pre-disposal sterilization. This proof-of-concept study highlights microwave technology as a potential approach for mitigating the risks of biofilm dissemination in clinical waste management, warranting further research to confirm its broader applicability.

### Microwave exposure reduces biofilm burden on catheter-mimic

**Figure 6:**
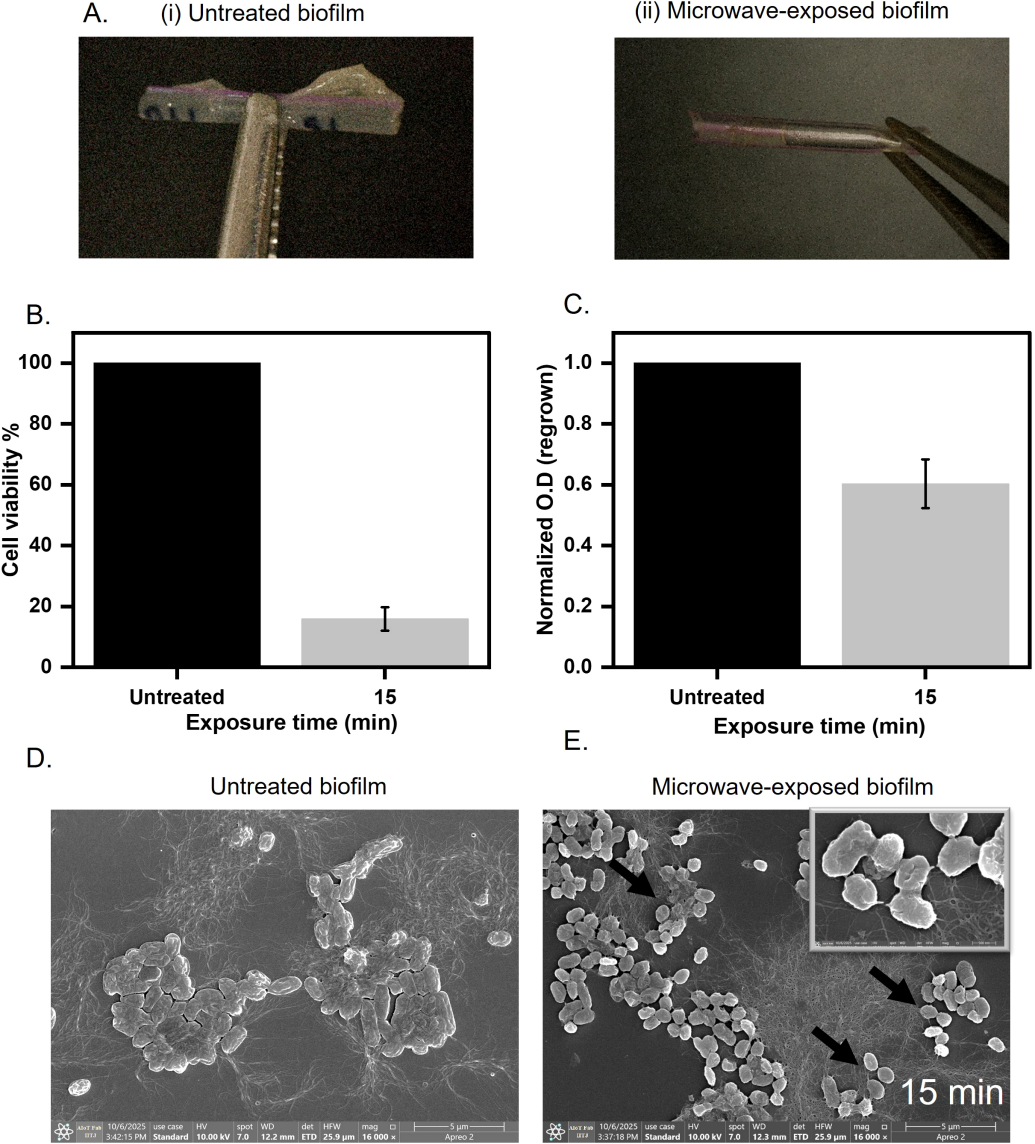
Biofilm grown over catheter mimic tube **(A)** Untreated and treated biofilms grown on the surface of the catheter mimic for 15 minutes at 0.4 W/cm^2^. **(B)** Cell viability assessment of microwave-exposed biofilm at 0.4 W/cm^2^ for 15 minutes on the catheter mimic. **(C)** Regrown capacity quantified by optical density at 600 nm after regrowing for 8 hours in LB broth at 37°C. **(D, E)** Untreated and microwave exposed biofilm was imaged under FE-SEM to visualize changes in cell structure and matrix morphology.

The significant disruption of biofilms due to microwave exposure prompted us to ask if the microwave radiation could also be used to remove biofilms from the catheter. Conventional methods, such as chemical sterilization (e.g., chlorhexidine) or ultrasonic scraping, often fail to penetrate dense biofilm architectures or risk damaging catheter substrates (e.g., silicone deformation under high heat) (Nishikawa et al., 2010; Bonez et al., 2013). In contrast, microwaves generate localized dielectric heating, which rises ~28°C (**Supplementary Figure 5A**), which selectively targets the matrix without compromising the catheter. To assess the effectiveness of microwave irradiation on biofilm-contaminated medical devices, we established *E. coli* UTI89 biofilm on a catheter mimic and subjected it to microwave exposure at 0.4 W/cm^2^ for 15 minutes (**Figure 6A**). Following treatment, metabolic activity, as measured by the MTT assay, showed a significant reduction, with cell viability decreasing by ~80% compared to the untreated biofilm (**Figure 6B****).** The biofilm biomass was also quantified after 15 minutes of exposure at 0.4 W/cm^2^, using crystal violet staining. We observed a ~50% reduction in biomass compared to the untreated cells (**Supplementary Figure 5B**). Furthermore, regrowth assays conducted in nutrient-rich media demonstrated a 50% decrease in the ability of the treated biofilms to recover and proliferate after irradiation (**Figure 6C****).** These results indicate that microwave treatment at the specified parameters effectively compromises both the viability and the regrowth potential of biofilm-associated bacteria on catheter surfaces. Next, we performed FESEM to visualize the morphological changes induced by microwave treatment in *E. coli* UTI89 biofilms established on catheter surfaces. In untreated samples, the biofilms exhibited a well-organized architecture, with rod-shaped *E. coli* cells surrounded by a cohesive layer of extracellular polymeric substances (EPS). In contrast, samples subjected to microwave irradiation exhibited pronounced cellular damage; the biofilm displayed a disrupted morphology, with cells showing membrane perforations and transitioning to collapsed ovoid forms. These changes indicate the loss of structural integrity and highlight the impact of microwave treatment on both cell and matrix ultrastructure (**Figures 6D, E**). An immersion leak test was performed to further assess the structural integrity of the catheter-mimic tube following 15 minutes of microwave exposure. The treated tube was sealed at one end with parafilm, while the opposite end was attached to a syringe. The tube was then submerged in water, and air was gradually purged into its lumen through the syringe. Throughout this procedure, the emergence of air bubbles at the tube’s surface was carefully monitored, as bubbling indicated the presence of leaks or surface defects induced by the microwave treatment. The absence of air bubbles confirmed that the catheter material remained intact, with no detectable loss of surface continuity or leakage under the tested conditions (**Supplementary Figure 5C**). This approach allowed for a rapid and practical evaluation of post-treatment material integrity, supporting the conclusion that microwave exposure did not result in observable damage to the catheter substrate. This highlights the potential of microwave irradiation as a practical and non-destructive approach for pre-disposal sterilization of biofilm-laden catheters, offering a promising alternative to conventional chemical or thermal disinfection methods that may cause damage to sensitive medical devices.

## Discussion

Biofilms pose a persistent challenge in clinical settings due to their enhanced resistance to antibiotics, disinfectants, and host immune responses (Liu et al., 2024). Hospital waste often harbours biofilm-contaminated materials that, if not properly sterilized, can serve as reservoirs for nosocomial pathogens and drive antimicrobial resistance (Perez-Bou et al., 2024). An Effective device sterilization is essential to prevent biofilm-associated hospital-acquired infections, yet current methods face challenges due to protocol lapses and inherent technological limitations. In contrast, microwave-based disinfection offers a promising alternative due to its energy efficiency, rapid action, low thermal loss, minimal environmental impact, and absence of toxic residues (Wang et al., 2020). While high-power microwaves have previously been explored for microbial sterilization, the risk of substrate degradation, particularly for heat-sensitive materials, has limited clinical translation (Banik et al., 2003; Park et al., 2017; Wang et al., 2024).

Our study investigated microwave irradiation (0.4 W/cm^2^ for 10–15 minutes) as a targeted strategy to disrupt biofilms on cover slips and catheter mimics. Our protocol achieved microbial inactivation in biofilm with preserved substrate integrity, marking a significant advancement in biofilm-safe sterilization. We established the minimum required radiation intensity and exposure time to disrupt biofilms. In many situations, the area to be treated is not large, and lower power is sufficient to maintain the optimum intensity of radiation required to treat the bacterial biofilm in a confined space. We observed a temperature elevation upon microwave radiation treatment, with a maximum temperature rise of 55°C at an intensity of 0.4 W/cm² for 15 minutes (~Δ18°C after 10 minutes and ~Δ34°C after 15 minutes; **Supplementary Figure 2A**). This aligns with findings by (Shamis et al., 2011), who demonstrated that microwave-induced thermal gradients disrupt microbial membrane integrity in *E. coli* UTI89 cells. Studies by *Li* et al. corroborate that sustained exposure to temperatures induces bacterial cell death by disrupting protein folding and metabolic pathways (Li et al., 2020). Consistent with this, our MTT assay revealed a decline in metabolic activity post-treatment. The suppression of metabolic activity suggests that microwave exposure effectively disrupts energy synthesis and impairs bacterial proliferation. Beyond immediate viability, a critical measure of biofilm disruption is the ability of surviving bacteria to regrow. Microwave exposure to *E. coli* UTI89 biofilm showed a reduction in regrowth capacity, indicating sublethal damage that impairs long-term recovery (**Figure 2C**). This data is significant because persister cells within biofilms often evade antimicrobial stress and resume growth after treatment cessation (Hall and Mah, 2017). A unique contribution of our study lies in the detailed analysis of EPS matrix integrity. Using ANS fluorescence, we detected a three-fold increase in hydrophobicity, indicative of exposure of hydrophobic domains in the microwave-damaged matrix. FE-SEM and CLSM revealed membrane perforation and matrix fragmentation, suggesting selective disruption of non-covalent matrix bonds without enzymatic degradation (**Figures 3A, B**). Ruby Red staining further confirmed altered protein distribution, pointing to destabilization of protein polymers essential for biofilm structure (**Figure 3C**). Additionally, an increase in extracellular DNA content indicates microwave-induced cell lysis and eDNA release, further weakening the matrix. As eDNA serves as a structural and regulatory scaffold within biofilms, its dispersal represents a critical mechanism for biofilm collapse (Flemming et al., 2016) (**Figure 4C**).

We compared our microwave protocol to UV disinfection, a commonly used method in hospital and industrial sterilization. Our findings showed that the UV-treated *E. coli* UTI89 biofilm retained 98% of its metabolic activity and exhibited no significant reduction in regrowth capacity (**Figure 5A**). This is consistent with EPS-mediated UV shielding and the ability of bacterial enzymes to repair UV-induced DNA damage (de Carvalho, 2017; Jones and Baxter, 2017). In contrast, microwave treatment achieved multi-tiered biofilm disruption, targeting both microbial viability and matrix cohesion, underscoring its efficacy for biofilm eradication. We established *E. coli* UTI89 biofilms on catheter mimics, materials frequently used in healthcare and highly susceptible to biofilm colonization. Our findings demonstrate that microwave irradiation effectively reduces both metabolic activity and regrowth potential without compromising the structural integrity of the catheter substrate (**Figures 6B, C****).** This presents a critical advantage over traditional high-power microwave systems, which, as noted by Banik et al., can cause thermal deformation of heat-sensitive polymers (Banik et al., 2003). The ability to sterilize without damaging the underlying material enhances the feasibility of integrating microwave-based protocols into routine hospital workflows, especially for the pre-disposal treatment of contaminated devices. Our study employed a single *E. coli* strain as a simplified model, acknowledging that biofilms present in hospital waste and clinical environments are typically multispecies and more complex. To fully understand the efficacy and limitations of microwave-based biofilm disruption, future investigations should incorporate clinically relevant multispecies biofilms, as well as perform more detailed analyses of biomass coverage and biofilm thickness. Such measurements would help clarify both the spatial extent and structural changes induced by microwave treatment. Furthermore, our method provides a non-chemical, residue-free alternative to conventional disinfection, aligning with the efforts of the WHO (World Health Organization) and CDC (Centers for Disease Control and Prevention) to develop sustainable, scalable, and low-cost infection control strategies in both high- and low-resource settings (Rutala and Weber, 2015). Although our results (**Supplementary Figure 5C**) indicate minimal substrate damage, the long-term effects of repeated microwave exposure on material properties have not been evaluated. However, this will be necessary to draw robust conclusions about clinical applicability and substrate durability, which warrant further investigation. Overall, our findings suggest that microwave radiation can effectively sterilize biofilm-laden surfaces without damaging underlying polymers, making the method viable for pre-disposal treatment of clinical waste. Importantly, early-stage matrix disruption was observed to dominate at shorter exposures, indicating that brief treatments can effectively disrupt biofilm integrity while minimizing energy consumption.

Our work contributes to a growing body of research on alternative sterilization strategies by offering a matrix-targeted, non-chemical approach that balances efficacy, safety, and cost. Future investigations should explore scalability across polymicrobial biofilms and different clinical pathogens, as well as optimize microwave parameters for integration with hybrid sterilization systems, including enzymatic and nanoparticle adjuncts.

## Conclusion

The observed suppression of metabolic activity and regrowth capacity in biofilms following microwave exposure suggests its potential in the pre-disposal treatment of contaminated medical devices. The reduction in viable biomass and diminished recovery of post-treatment biofilms may contribute to lowering the risk of antimicrobial resistance (AMR) dissemination from clinical waste. These findings suggest that microwave exposure impacts both cellular viability and biofilm matrix integrity, providing a non-chemical, energy-efficient approach that could complement existing decontamination strategies. By identifying the minimum exposure time and intensity required for effective biofilm disruption, this study provides preliminary parameters that could inform the development of microwave-based systems. Such systems may allow for optimized power usage, potentially reducing operational costs while maintaining efficacy. The relatively rapid action and scalability of microwave treatment make it a suitable option for further exploration in hospital waste management settings, particularly where efficient, chemical-free sterilization methods are required. However, further validation under real-world conditions would be necessary to assess its practical integration into clinical workflows and to evaluate long-term performance across different device types and microbial species.

## Data Availability

The original contributions presented in the study are included in the article/**Supplementary Material**. Further inquiries can be directed to the corresponding authors.
