# Supplementary material for: Breaking the barrier: disruption of bacterial biofilms using microwave radiation
Source: Front Cell Infect Microbiol. 2025 Nov 18;15:1670237. doi: 10.3389/fcimb.2025.1670237 (PMC12669098; doi:10.3389/fcimb.2025.1670237)
Supplement: Supplementary file 1 [file Table1.docx]

**Supplementary Information**

**Breaking the Barrier: Disruption of Bacterial Biofilms Using Microwave Radiation**

Harita Ben^1^, Harshita Agarwal^1^, Bharat Gurnani^2^, Aman A. Pradhan^3^, Arani Ali Khan^3*^ and Neha Jain^1*^

^1^Department of Bioscience and Bioengineering, Indian Institute of Technology, Jodhpur, Karwar, 342030, Rajasthan, India

^2^Centre of Excellence-AyurTech, Indian Institute of Technology, Jodhpur, Karwar, 342030, Rajasthan, India

^3^Department of Electrical Engineering, Jodhpur, Karwar, 342030, Rajasthan, India

^*^Corresponding author emails: aakhan@iitj.ac.in and njain@iitj.ac.in

Supplementary Figure 1 Optimization of different microwave powers 0.2 W/cm^2^, 0.4 W/cm^2^, and 1 W/cm^2^) and different exposure times (10-60 minutes) to disinfect *E. coli* biofilm grown over a coverslip in YESCA + 4% DMSO broth for four days at 25 ℃.

Supplementary Figure 2 (A) The change in temperature upon exposure for 10 and 15 minutes was monitored by thermal gun at 0.4 W/cm^2^. (B) Quantification of biofilm biomass without and with microwave exposure at 0.4 W/cm^2^ for 10- and 15-minutes using crystal violet stain. (C) Effect of conventional method (dry heat) on the cell viability of biofilm after treatment of 10 minutes at 45 ⁰C and 15 min at 56 ⁰C.

Supplementary Figure 3 Representative FE-SEM images of samples prepared without sonication from A (i) untreated and B (ii) microwave-treated biofilms for 10 and 15 minutes at 0.4 W/cm^2^.

Supplementary Figure 4 Comparison of heat-induced hydrophobicity of biofilm matrix upon 20 minutes UV and microwave exposure estimated by ANS dye.

Supplementary Figure 5 (A) The change in temperature of biofilm grown over a catheter mimic, upon microwave exposure for 10 and 15 minutes, was monitored by thermal gun. (B) Quantification of biofilm biomass on catheter mimic without and with microwave exposure at 0.4 W/cm^2^ for 15-minutes using crystal violet stain. (C) Evaluating material integrity of catheter mimic after microwave exposure by purging air (i) without biofilm and (ii) with biofilm.
